# A randomized controlled trial of enhancing hypoxia-mediated right cardiac mechanics and reducing afterload after high intensity interval training in sedentary men

**DOI:** 10.1038/s41598-021-91618-0

**Published:** 2021-06-15

**Authors:** Yu-Chieh Huang, Chih-Chin Hsu, Tieh-Cheng Fu, Jong-Shyan Wang

**Affiliations:** 1 Department of Physical Therapy, College of Medical and Health Science, Asia University, Taichung, Taiwan; 2 Heart Failure Center, Department of Physical Medicine and Rehabilitation, Keelung Chang Gung Memorial Hospital, Keelung, Taiwan; 3 Healthy Aging Research Center, Graduate Institute of Rehabilitation Science, Medical Collage, Chang Gung University, Taoyuan, Taiwan; 4 Research Center for Chinese Herbal Medicine, College of Human Ecology, Chang Gung University of Science and Technology, Taoyuan, Taiwan

**Keywords:** Cardiovascular biology, Circulation, Cardiovascular biology, Disease prevention, Medical imaging

## Abstract

Hypoxic exposure increases right ventricular (RV) afterload by triggering pulmonary hypertension, with consequent effects on the structure and function of the RV. Improved myocardial contractility is a critical circulatory adaptation to exercise training. However, the types of exercise that enhance right cardiac mechanics during hypoxic stress have not yet been identified. This study investigated how high-intensity interval training (HIIT) and moderate-intensity continuous training (MICT) influence right cardiac mechanics during hypoxic exercise A total of 54 young and healthy sedentary males were randomly selected to engage in either HIIT (3-min intervals at 40% and 80% of oxygen uptake reserve, n = 18) or MICT (sustained 60% of oxygen uptake reserve, n = 18) for 30 min/day and 5 days/week for 6 weeks or were included in a control group (CTL, n = 18) that did not engage in any exercise. The primary outcome was the change in right cardiac mechanics during semiupright bicycle exercise under hypoxic conditions (i.e., 50 watts under 12% FiO_2_ for 3 min) as measured by two-dimensional speckle tracking echocardiography.**:** After 6 weeks of training, HIIT was superior to MICT in improving maximal oxygen consumption (VO_2max_). Furthermore, the HIIT group showed reduced pulmonary vascular resistance (PVR, pre-HIIT:1.16 ± 0.05 WU; post-HIIT:1.05 ± 0.05 WU, p < 0.05) as well as an elevated right ventricular ejection fraction (RVEF, pre-HIIT: 59.5 ± 6.0%; post-HIIT: 69.1 ± 2.8%, p < 0.05) during hypoxic exercise, coupled with a significant enhancement of the right atrial (RA) reservoir and conduit functions. HIIT is superior to MICT in dilating RV chamber and reducing radial strain but ameliorating radial strain rate in either systole (post-HIIT: 2.78 ± 0.14 s^-1^; post-MICT: 2.27 ± 0.12 s^-1^, p < 0.05) or diastole (post-HIIT: − 2.63 ± 0.12 s^-1^; post-MICT: − 2.36 ± 0.18 s^-1^, p < 0.05). In the correlation analysis, the changes in RVEF were directly associated with improved RA reservoir (r = 0.60, p < 0.05) and conduit functions (r = 0.64, p < 0.01) but inversely associated with the change in RV radial strain (r = − 0.70, p < 0.01) and PVR (r = − 0.70, p < 0.01) caused by HIIT. HIIT is superior to MICT in improving right cardiac mechanics by simultaneously increasing RA reservoir and conduit functions and decreasing PVR during hypoxic exercise.

## Introduction

According to the Frank–Starling mechanism, the ventricle increases its contractility and stroke volume in response to the augmented blood return and hence preload. However, the Frank–Starling curve is disturbed by the changed condition of afterload or inotropy^1^. Hypoxic exposure induces pulmonary vasoconstriction and hypertension, increases right ventricular (RV) afterload, and induces changes in RV function and dimension^2^; consequently, it limits physical performance during exercise in healthy individuals and patients with respiratory diseases^3^. In this study, we used a novel methodology combining both acute hypoxic exposure and exercise stress to generate perturbations to the RV.

Conventionally, it was considered that the RV is exposed to volume overload only due to increased cardiac output (CO) during exercise; nevertheless, a recent study further demonstrated the presence of significant pressure overload^4^. Furthermore, significant volume overload may cause enlargement of the RV and right atrium (RA) with a relatively enhanced longitudinal function in the RV^5^, whereas RV pressure overload primarily affects radial shortening and induces changes in fiber orientation in the RV^6^. Although most studies have focused on the longitudinal motion of the RV, current data suggest that radial motion seems to be a key indicator to assess RV pump function as well^7^. In the concept of mixed hemodynamic RV overload in exercise, these effects can be distinct in different exercise regimens and may also induce functional remodeling. However, few studies have elucidated the distinct effects of different training regimens on radial or longitudinal RV mechanics.

Endurance training is a valuable approach to the management of pulmonary-related disease by augmenting pulmonary vasodilatation^8^. Multiple studies indicate greater favorable cardiovascular adaptations after exercise is performed at higher intensity than low or moderate levels for healthy subjects^9^, left ventricular dysfunction^10^, or pulmonary-related disease^11^. Because of the larger venous return and more blood fills into RV, HIIT is superior to the MICT in prompting cardiac remodeling in either left or right chamber dimension^12^. These structural alterations represent cardiac adaptations to the high hemodynamic demands of exercise and may further influence the cardiac mechanics. Furthermore, in a rat model of pulmonary hypertension, only HIIT lowered pulmonary vascular resistance (PVR), suppressed RV hypertrophy and prompted higher cardiac index and apelin expression (a potent inotropic substance), which may be explained by more satisfactory for pulmonary vascular endothelial adaptation to the HIIT pulsatile stimulus^13^. However, although major studies have focused on the left heart, only a few human studies have focused on the right heart in association with HIIT or MICT. An effective training strategy that could enhance right cardiac mechanics during hypoxic exercise has not yet been established.

Typically, right cardiac performance is assessed by echocardiography under normoxic environments, which only displays chamber size and wall motion by two-dimensional speckle-tracking echocardiography (2D-STE) with normal estimated PVR^14^. To comprehensively explore the distinctive mechanical responses to mixed hemodynamic RV overload following various exercise interventions, exercise stress echocardiography^15^ with 2D-STE was performed in a 12% FiO_2_ hypoxic environment.

In this study, we hypothesize that acute hypoxic exercise would provoke volume load to enhance RV contractility in longitudinal motion, whereas after 6 weeks of interventions, HIIT, resulting in a significantly dilated RV, would be superior to MICT in diminishing PVR and enhancing RA functions, which further affects the RV in both radial and longitudinal motions. This study aimed to compare the effectiveness of HIIT and MICT in improving right cardiac mechanics during hypoxic exercise. It comprehensively clarified how HIIT (3-min intervals at 40% and 80% of oxygen consumption reserve or MICT (sustained 60% of oxygen consumption reserve) for 6 weeks affected right cardiac mechanics at rest or during hypoxic exercise in sedentary male using 2D-STE.

## Results

### Cardiopulmonary fitness in normoxia

To assess the cardiopulmonary fitness, the cardiopulmonary exercise test (CPET) was performed in a normoxic condition. No significant difference in anthropometric parameters or functional capacity among the three groups was found at the onset of the study (Table 1). All subjects had no altered trend in dietary records or original physical activity (data not shown), and the CTL group kept in sedentary lifestyle without receive any other exercise training (data not shown). After the six-week interventions, HIIT significantly increased stroke volume (SV) and decreased mean arterial pressure and total peripheral resistance (TPR) at rest in normoxic conditions (*P* < 0.05, Table 1). Moreover, either HIIT or MICT demonstrated improved cardiopulmonary fitness by increased work rates, respiratory minute volume (V_E_), and oxygen consumption (VO_2_) at the ventilation threshold and peak exercise performance (*P* < 0.05, Table 1). However, HIIT led to a greater improvement in aerobic capacity (such as VO_2max_) than MICT (*P* < 0.05, Table 1). No significant changes in cardiopulmonary responses to the CPET were observed after 6 weeks in the CTL group (Table 1).

**Table 1:** The effects of interval and continuous exercise regimens on cardiopulmonary fitness in normoxia.

	HIIT		MICT		CTL	
	Pre	Post	Pre	Post	Pre	Post
*Anthropometric data*						
Age, yr	21.1 ± 0.4	–	21.3 ± 0.6	–	21.9 ± 0.5	–
Height, cm	172.1 ± 1.1	–	172.9 ± 0.6	–	173.0 ± 0.9	–
Weight, kg	67.6 ± 2.0	68.1 ± 1.7	67.1 ± 1.4	67.0 ± 1.4	66.2 ± 1.9	65.8 ± 1.7
BMI, kg·m^−2^	22.5 ± 0.4	22.5 ± 0.4	22.3 ± 0.7	22.4 ± 0.5	21.9 ± 0.5	22.0 ± 0.9
*Hemodynamic characteristics at rest*						
HR, bpm	69 ± 2	67 ± 1	69 ± 2	67 ± 2	69 ± 3	69 ± 4
SV, ml	68.5 ± 2.6	78.3 ± 1.4 *,^#^	68.6 ± 2.7	75.2 ± 2.1	68.2 ± 2.0	68.5 ± 2.3
CO, L·min^−1^	4.8 ± 0.3	5.1 ± 0.2	4.8 ± 0.4	5.0 ± 0.4	4.7 ± 0.8	4.8 ± 0.5
MAP, mmHg	90 ± 2	85 ± 2 *	91 ± 3	86 ± 2 *	88 ± 3	89 ± 3
TPR, mmHg·L^−1^·min^−1^	18.7 ± 0.7	16.5 ± 0.8 *	19.1 ± 0.6	17.0 ± 0.7 *	18.7 ± 0.9	18.5 ± 1.1
*Ventilatory threshold*						
Work-rate, W	108 ± 9	157 ± 6 *,**,^#^	109 ± 6	137 ± 7 *,^#^	106 ± 7	108 ± 11
HR, bpm	137 ± 6	156 ± 5 *,**,^#^	136 ± 5	146 ± 4 *	138 ± 6	139 ± 8
VO_2_, mL·min^−1^·kg^−1^	20.8 ± 1.1	27.3 ± 1.2 *,**,^#^	21.1 ± 1.6	23.9 ± 1.1 *	21.5 ± 1.2	21.2 ± 1.4
*Maximal performance*						
Work-rate, W	189 ± 8	241 ± 6 *,**,^#^	190 ± 6	226 ± 7 *,^#^	195 ± 8	201 ± 6
HR, bpm	196 ± 3	197 ± 3	193 ± 5	197 ± 3	195 ± 4	196 ± 2
VO_2_, mL·min^−1^·kg^−1^	34.5 ± 1.3	46.8 ± 2.1 *,**,^#^	35.1 ± 1.2	41.6 ± 1.9 *,^#^	34.6 ± 1.3	35.1 ± 1.0

Values were mean ± SEM. Data of hemodynamic characteristics at rest were acquired from the subjects who rested for 5 min on a bicycle. HIIT, high-intensity interval training group; MICT, moderate-intensity continuous training group; CTL, control group; Pre, pre-intervention; Post, post-intervention; BMI, body mass index; HR, heart rate; SV, stroke volume; CO, cardiac output; MAP, mean arterial pressure; TPR, total peripheral pressure; VO_2_, oxygen consumption. * P < 0.05, Pre vs*.* Post; ** P < 0.05, HIIT vs. MICT; # p < 0.05, HIIT or MICT vs CTL.

### Conventional echocardiographic parameters in hypoxia

Under hypoxic conditions, both HIIT and MICT simultaneously lowered TPR and PVR at rest; moreover, only HIIT further decreased RV afterload, PVR and right ventricular systolic pressure (RVSP) during hypoxic exercise (*P* < 0.05, Table 2). Although both types of training augmented the peak velocity of early tricuspid blood flow, HIIT was superior to MICT. Furthermore, only HIIT significantly elevated right ventricular ejection fraction (RVEF) (*P* < 0.05, Table 2). No significant changes in echocardiographic parameters were observed after 6 weeks in the CTL group (Table 2).

**Table 2:** The effects of interval and continuous exercise regimens on echocardiographic parameters in hypoxia at rest and during HE**.**

	HIIT		MICT		CTL	
	Pre	Post	Pre	Post	Pre	Post
*Heart Rate*	76 ± 2	72 ± 3	75 ± 2	74 ± 2	75 ± 3	75 ± 2
*Systemic Resistance*						
MAP, mmHg	81 ± 2	76 ± 3 *, #	81 ± 3	74 ± 3 *, #	82 ± 3	82 ± 2
TPR, mmHg·L^−1^·min^−1^	22.8 ± 1.5	18.4 ± 1.3 *, #	21.7 ± 1.2	18.7 ± 1.4 *, #	22.1 ± 0.9	22.3 ± 1.2
RPP, mmHg·bpm	81.3 ± 2.1	75.9 ± 2.5 *, #	81.1 ± 2.7	74.0 ± 2.8 *, #	82.1 ± 3.5	82.4 ± 2.0
*Pulmonary Resistance*						
PVR rest, WU	1.02 ± 0.04	0.84 ± 0.03 *, #	1.05 ± 0.08	0.90 ± 0.04 *	1.01 ± 0.04	1.04 ± 0.07
PVR ex, WU	1.16 ± 0.05	1.05 ± 0.05 *, #	1.17 ± 0.05	1.13 ± 0.06	1.17 ± 0.04	1.17 ± 0.03
RVSP rest, mmHg	27.0 ± 1.3	21.1 ± 1.2 *, #	27.6 ± 2.2	22.8 ± 1.2 *, #	27.2 ± 1.4	27.9 ± 1.9
RVSP ex, mmHg	29.4 ± 1.6	23.2 ± 1.1 *, #	29.6 ± 2.2	26.5 ± 1.3	29.2 ± 1.6	29.8 ± 1.9
*Right Ventricular Systolic Function*						
RVEF, %	59.5 ± 6.0	69.1 ± 2.8 *	60.5 ± 3.5	62.4 ± 3.8	61.2 ± 3.1	62.8 ± 5.9
RVOT VTI, cm^2^	0.28 ± 0.01	0.31 ± 0.01 *	0.28 ± 0.02	0.30 ± 0.01	0.29 ± 0.02	0.28 ± 0.02
TAPSE, cm	2.17 ± 0.06	2.12 ± 0.04	2.17 ± 0.07	2.19 ± 0.07	2.17 ± 0.05	2.17 ± 0.09
*RA pressure*						
IVC diameter, cm	1.54 ± 0.05	1.53 ± 0.04	1.56 ± 0.05	1.53 ± 0.04	1.53 ± 0.09	1.52 ± 0.06
*Tricuspid velocity (TV)*						
E, m/s	1.79 ± 0.10	2.18 ± 0.11 *, #	1.78 ± 0.09	2.02 ± 0.10 *	1.77 ± 0.1	1.78 ± 0.1
E', m/s	0.158 ± 0.006	0.164 ± 0.006	0.156 ± 0.006	0.161 ± 0.004	0.159 ± 0.012	0.157 ± 0.01
E/E', ratio	11.26 ± 0.71	13.49 ± 0.77	11.64 ± 0.82	12.18 ± 0.50	11.33 ± 1.08	11.63 ± 1.06

Values were mean ± SEM. Resting images were acquired after the subject was placed in the semi-supine position for 10 min under hypoxia. HIIT, high-intensity interval training group; MICT, moderate-intensity continuous training group; CTL, control group; Pre, pre-intervention; Post, post-intervention; MAP, mean arterial pressure; TPR, total peripheral pressure; RPP, rate pressure product; PVR, pulmonary vascular resistance; RVSP, right ventricular systolic pressure; RVEF, right ventricular ejection fraction; RVOT VTI, right ventricular outflow tract velocity time integral; TAPSE, tricuspid annular plane systolic excursion; IVC, inferior vena cava; E, the peak velocity of early tricuspid blood flow; E’, the peak velocity of early tricuspid annular tissue; * P < 0.05, Pre vs*.* Post; ** P < 0.05, HIIT vs. MICT; # p < 0.05, HIIT or MICT vs CTL.

### Right atrium (RA) functions

**Figure 1:**
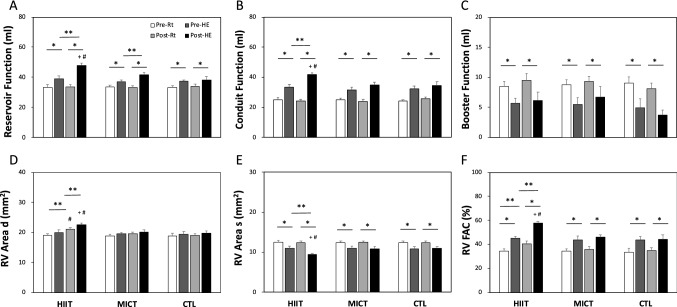
Comparisons of the effects of various exercise regimens on RA volumes and RV area parameters at rest or during HE. (**A**) Reservoir function; (**B**) Conduit function; (**C**) Booster function; (**D**) RV area in end-diastole; (**E**) RV area in end-systole; (**F**) RV fractional area change (FAC). HIIT, high-intensity interval training group; MICT, moderate-intensity continuous training group; CTL, control group; Pre-Rt, resting before the intervention; Pre-HE, during HE before the intervention; Post-Rt, resting after the intervention; Post-HE, during HE after the intervention. Values are the mean ± SEM. * *P* < 0.05, Rt vs. HE; ** *P* < 0.05, Pre vs. Post; + *P* < 0.05, HIIT vs. MICT; # *P* < 0.05, HIIT or MICT vs CTL.

Before interventions, acute hypoxic exercise increased reservoir and conduit volumes and decreased the booster volume in the RA (*P* < 0.05, Fig. 1A–C). Following the 6-week interventions, HIIT induced increases in RA reservoir (Fig. 1A) and conduit volumes (Fig. 1B), while MICT ameliorated only RA reservoir volume during hypoxic exercise. No changes in RA volumes occurred at rest or during hypoxic exercise after 6 weeks in the CTL group (Fig. 1A–C).

### Area and dimensions of the right ventricle under hypoxia

**Figure 2:**
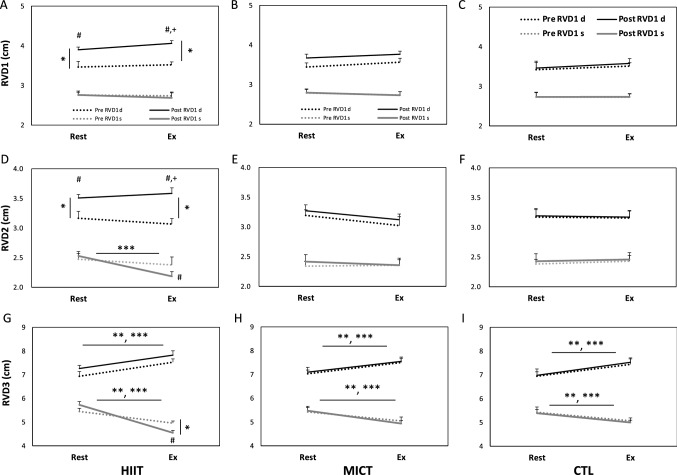
Comparisons of the effects of various exercise regimens on RV dimensions at rest or during HE. **(A)** RVD1 in HIIT; **(B)** RVD1 in MICT; **(C)** RVD1 in CTL; (**D)** RVD2 in HIIT; **(E)** RVD2 in MICT; **(F)** RVD2 in CTL; (**G**) RVD3 in HIIT; (**H**) RVD3 in MICT; (**I**) RVD3 in CTL. HIIT, high-intensity interval training group; MICT, moderate-intensity continuous training group; CTL, control group; Pre RVD d, RVD in end-diastole before training; Post RVD d, RVD in end-diastole after training; Pre RVD s, RVD in end-systole before training; Post RVD s, RVD in end-systole after training. Values are the mean ± SEM. * *P* < 0.05, Pre vs. Post; ** *P* < 0.05, Pre-Rt vs. Pre-HE; *** *P* < 0.05, Post-Rt vs. Post-HE; + *P* < 0.05, HIIT vs. MICT; # *P* < 0.05, HIIT or MICT vs CTL.

Figure 2 further shows details of the dimensions of the RV at rest and during hypoxic exercise. A bout of acute hypoxic exercise augmented RV fractional area change (FAC%, Fig. 1F) by the reducing systolic RV area (Fig. 1D–F) which was not depend on radial diameters (RVD1 or RVD2) (Fig. 2A–F) but RV longitudinal diameter (RVD3) (Fig. 2G–I). Following the 6-week intervention, HIIT was superior to MICT in enhancing FAC% by a greater diastolic RV area (Fig. 1D–F). Meanwhile, a significantly enlarged RV was observed in the HIIT group, as demonstrated by a longer diastole RV basal diameter (RVD1) (Fig. 2A–C) and middle diameter (RVD2) (Fig. 2D–F), when compared to the MICT and CTL groups. Moreover, compared with no exercise, HIIT led to shorter systoles in both RVD2 and RVD3 during hypoxic exercise. (P < 0.05, Fig. 2D,G).

### Strain and strain rate in the RA and RV under hypoxia

To investigate the right cardiac mechanics under acute hypoxic exposure, the exercise stress echocardiography using 2D-STE technique was performed and the imaging was collected at both resting and during exercise. Before the interventions, acute hypoxic exercise enhanced both RA radial and longitudinal strain and strain rate (*P* < 0.05, Table 3). In addition, in the RV, both longitudinal strain and strain rate were increased while reducing radial strain under hypoxic exercise (*P* < 0.05, Table 4). Following the 6-week interventions, both HIIT and MICT augmented RA radial strain and systolic/diastolic strain rate at rest or during hypoxic exercise, whereas only HIIT increased RA longitudinal strain and systolic strain rate during hypoxic exercise (*P* < 0.05, Table 3). On the other hand, although HIIT, but not MICT, decreased RV radial strain, the systolic/diastolic strain rate was ameliorated (*P* < 0.05, Table 4). Moreover, no significant changes were found in radial/longitudinal strain and systolic/diastolic strain rate in the RA or RV at rest or during hypoxic exercise after 6 weeks in the CTL group (*P* < 0.05, Tables 3 and 4).

**Table 3:** The effects of interval and continuous exercise regimens on RA mechanics in HE**.**

	HIIT		MICT		CTL	
	Pre	Post	Pre	Post	Pre	Post
**RA strains and strain rates at rest and during exercise under 12% hypoxic exposure**						
*Radial Strain (%)*						
Rt	− 27.39 ± 3.08	− 35.09 ± 3.11 *	− 28.28 ± 2.36	− 35.65 ± 3.67 *	− 27.74 ± 3.95	− 29.39 ± 1.77
Ex	− 26.96 ± 5.62	− 47.49 ± 2.68 *,**, #	− 25.36 ± 4.36	− 41.63 ± 2.27 *,**, #	− 25.74 ± 4.72	− 27.71 ± 4.95
*Radial Strain Rate (s*^*-1*^*)*						
RA in Dia						
Rt	− 1.81 ± 0.11	− 2.28 ± 0.24 *	− 1.88 ± 0.13	− 2.68 ± 0.41 *	− 1.82 ± 0.19	− 1.93 ± 0.16
Ex	− 2.38 ± 0.26 **	− 3.28 ± 0.26 *,**, #	− 2.4 ± 0.29 **	− 3.61 ± 0.22 *,**, #	− 2.43 ± 0.26 **	− 2.33 ± 0.38 **
RA in Sys						
Rt	1.95 ± 0.16	2.49 ± 0.25 *	1.95 ± 0.15	2.54 ± 0.39 *	1.95 ± 0.08	1.96 ± 0.15
Ex	2.38 ± 0.11 **	3.3 ± 0.19 *,**, #	2.47 ± 0.29 **	3.43 ± 0.33 *,** #	2.39 ± 0.19 **	2.41 ± 0.17 **
*Longitudinal Strain (%)*						
Rt	45.06 ± 4.19	39.88 ± 3.81	46.65 ± 4.45	43.21 ± 2.54	45.76 ± 7.06	43.76 ± 4.57
Ex	59.32 ± 4.12 **	69.38 ± 3.17*,**,***	57.83 ± 5.05 **	54.01 ± 2.81 **	59.32 ± 4.12 **	59.87 ± 5.37 **
*Longitudinal Strain Rate (s-1)*						
RA in Dia						
Rt	1.67 ± 0.09	1.51 ± 0.08	1.66 ± 0.09	1.7 ± 0.12	1.66 ± 0.14	1.61 ± 0.12
Ex	2.59 ± 0.09 **	2.6 ± 0.14 **	2.56 ± 0.16 **	2.3 ± 0.17 **	2.56 ± 0.3 **	2.49 ± 0.33 **
RA in Sys						
Rt	− 1.74 ± 0.16	− 1.59 ± 0.13	− 1.77 ± 0.14	− 1.75 ± 0.13	− 1.8 ± 0.26	− 1.77 ± 0.16
Ex	− 2.94 ± 0.19 **	− 3.89 ± 0.28 *,**,***, #	− 2.96 ± 0.25 **	− 2.75 ± 0.18 **	− 2.95 ± 0.22 **	− 2.9 ± 0.34 **

Values were mean ± SEM. Values were mean ± SEM. RA in Dia, when RV in the systolic phase; RA in Sys, when RV in the diastolic phase. HIIT, high-intensity interval training group; MICT, moderate-intensity continuous training group; CTL, control group; Rt, at rest; Ex, during exercise; Post, postintervention; Pre, preintervention. *P < 0.05, Pre- vs Post-; **P < 0.05, Rt vs Ex; ***P < 0.05, HIIT vs MICT; # p < 0.05, HIIT or MICT vs CTL.

**Table 4:** The effects of interval and continuous exercise regimens on RV mechanics in HE.

	HIIT		MICT		CTL	
	Pre	Post	Pre	Post	Pre	Post
**RV strains and strain rates at rest and during exercise under 12% hypoxic exposure**						
*Radial Strain (%)*						
Rt	35.3 ± 2.5	26.7 ± 2.0 *	34.8 ± 2.7	31.8 ± 2.1	35.8 ± 3.0	34.7 ± 4.2
Ex	23.4 ± 2.2 **	18.5 ± 2.1 *,**	22.4 ± 3.9 **	21.6 ± 2.7 **	22.0 ± 7.6 **	20.0 ± 3.6 **
*Radial Strain Rate (s*^*-1*^*)*						
RV in Sys						
Rt	2.2 ± 0.19	2.78 ± 0.14 *, ***, #	2.15 ± 0.20	2.27 ± 0.12	2.12 ± 0.35	2.20 ± 0.31
Ex	2.13 ± 0.11	2.55 ± 0.18 *, ***, #	2.01 ± 0.24	2.21 ± 0.18	1.95 ± 0.20	2.02 ± 0.16
RV in Dia						
Rt	− 1.47 ± 0.22	− 2.63 ± 0.12 *, ***, #	− 1.54 ± 0.12	− 2.36 ± 0.18 *, #	− 1.53 ± 0.16	− 1.45 ± 0.2
Ex	− 2.06 ± 0.16 **	− 1.78 ± 0.21 **	− 1.98 ± 0.22 **	− 2.02 ± 0.18 **	− 2.02 ± 0.11 **	− 1.99 ± 0.24 **
*Longitudinal Strain (%)*						
Rt	− 19.54 ± 1.28	− 18.12 ± 1.02	− 18.13 ± 0.95	− 19.46 ± 0.93	− 18.61 ± 1.47	− 19.1 ± 1.45
Ex	− 25.58 ± 1.66 **	− 23.47 ± 3.31 **	− 23.3 ± 1.36 **	− 23.16 ± 1.68 **	− 24.39 ± 2.01 **	− 24.79 ± 1.27 **
*Longitudinal Strain Rate (s-1)*						
RV in Sys						
Rt	− 1.16 ± 0.06	− 1.10 ± 0.06	− 1.12 ± 0.1	− 1.17 ± 0.10	− 1.16 ± 0.09	− 1.17 ± 0.09
Ex	− 1.74 ± 0.22 **	− 1.72 ± 0.26 **	− 1.78 ± 0.12 **	− 1.70 ± 0.14 **	− 1.77 ± 0.16 **	− 1.82 ± 0.15 **
RV in Dia						
Rt	1.30 ± 0.10	1.25 ± 0.08	1.32 ± 0.11	1.27 ± 0.12	1.31 ± 0.13	1.30 ± 0.14
Ex	2.33 ± 0.18 **	2.19 ± 0.24 **	2.33 ± 0.16 **	2.17 ± 0.07 **	2.32 ± 0.13 **	2.34 ± 0.15 **

Values were mean ± SEM. Values were mean ± SEM. RV in Sys: when RV in systolic phase; RV in Dia: when RV in diastolic phase. *P < 0.05, Pre- vs Post- ; **P < 0.05, Rt vs Ex; ***P < 0.05, HIIT vs MICT; #p < 0.05, HIIT or MICT vs CTL.

### Relationship between hypoxic exercise-induced changes in right cardiac mechanical variables following interventions

**Figure 3:**
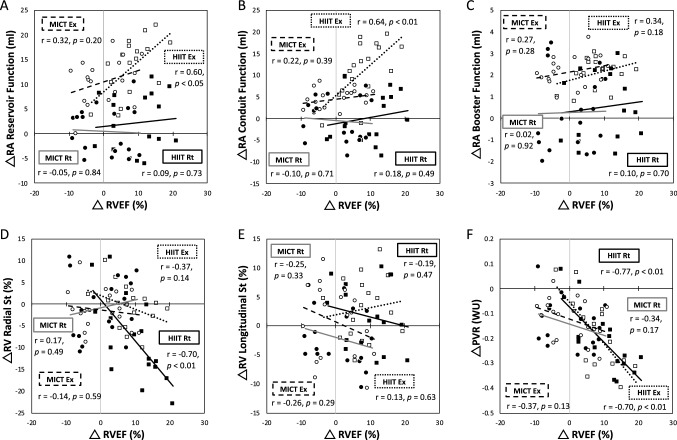
Correlations between changes in RVEF and RA functions, RV mechanics and PVR at rest or during HE following various interventions. RA, right atrium; RV, right ventricle; HIIT, high-intensity interval training group; MICT, moderate-intensity continuous training group; Ex, during HE; Rt, at rest. RVEF, right ventricle ejection fraction; St, strain; PVR, pulmonary vascular resistance. ●(closed circles), MICT Rt; ○ (open circles), MICT Ex; ■ (closed squares), HIIT Rt; □(open squares), HIIT Ex; Δ, postintervention minus preintervention. The solid line indicates the trend line of HIIT Rt; the dotted line indicates the trend line of HIIT Ex; the gray line indicates the trend line of MICT Rt; and the dashed line indicates the trend line of MICT Ex.

Here, the change indicates the difference between pretraining and posttraining. No significant resting relationship was observed between RA volumes and RVEF following either intervention (Fig. 3A–C). However, hypoxic exercise-induced changes in the RA reservoir (Fig. 3A; r = 0.60, P < 0.05) and conduit volumes (Fig. 3B; r = 0.64, P < 0.01) were positively associated with RVEF after HIIT. Furthermore, the hypoxic exercise-induced change in RV radial strain was negatively correlated with the RVEF change in the HIIT group (Fig. 3D; r =  − 0.70, P < 0.01). However, no significant correlations were found between hypoxic exercise-induced changes in RVEF and RV longitudinal strain following HIIT (Fig. 3E). Additionally, in the HIIT group, both resting (Fig. 3F; r =  − 0.77, P < 0.01) and hypoxic exercise-induced changes in PVR (Fig. 3F; r =  − 0.70, P < 0.01) were inversely associated with RVEF. In contrast to the HIIT group, no significant correlations were observed among RA reservoir, conduit, and booster pump functions; radial/longitudinal strains and strain rates in the RV; and PVR at rest or during hypoxic exercise in the MICT or CTL groups (Fig. 3).

### Test–retest reliability

The intraobserver intraclass correlation coefficient (ICC) and variability (CV%) were 0.89 (0.74 ~ 0.95) and 7.9 (7.6–8.2), respectively, for radial strain, whereas they were 0.93 (0.83 ~ 0.97) and 5.7 (5.2–6.2), respectively, for longitudinal data (see Supplementary Table S1 online). In addition, the Cronbach alpha value was 0.94 for radial motion and 0.95 for longitudinal motion. Supplementary Fig. S1 shows the Bland–Altman plots and correlation dot plots for descriptive purposes.

## Discussion

This is the first investigation to clarify the effects of various exercise regimens on right cardiac mechanics during hypoxic exercise using 2D-STE technology. Both HIIT and MICT improve RA reservoir function, while only HIIT enhances RA conduit function to reinforce RV preload. Therefore, HIIT is more efficient than MICT in dilating the chamber of the RV by ameliorating radial strain rate but reducing radial strain in either systole or diastole. Although both interventions lessen resting RV afterload, PVR and RVSP, only HIIT further diminishes PVR under hypoxic exercise. Notably, the correlation analysis further demonstrated that an augmented RVEF is significantly associated with greater RA reservoir and conduit functions and lower PVR following HIIT.

The atrium tends to dilate in response to greater venous return or chronic elevations in ventricular filling pressure when exercising^16^. However, the elevated RA afterload caused by acute hypoxic exposure may cause the ratio of passive reservoir to active contraction to decline^17^. In our longitudinal study, both HIIT and MICT for 6 weeks enhanced reservoir function even during hypoxic exercise to accommodate more blood return. These results correspond with a cross-sectional investigation, i.e., highly dynamic athletes had larger RA reservoir functions for venous return and more blood filling into the RV than less dynamic athletes^18^. As the enhanced peak velocity of early tricuspid blood flow represented, the ameliorated conduit function in HIIT accelerated RV early filling and enhanced RV preload even under hypoxic stress.

Because of the higher cardiac output demand in the HIIT, volume load-related remodeling may be increased in the HIIT group compared with that in the MICT group^19^. Although most studies have focused on longitudinal motion to generate RV ejection, our study further confirmed that RV dilation primarily occurred in the radial direction instead of the longitudinal direction, which was consistent with the unchanged tricuspid annular plane systolic excursion (TAPSE) in both interventions. Elevated radial motion influences systolic function via the bellows effect because the free wall of the RV has a larger surface than the tricuspid annular cross-sectional area^20^. Therefore, we speculated that the improvement of RVEF during hypoxic exercise is related with not only longitudinal but also radial motion.

Although TAPSE is a widely utilized parameter for RV function with an M-mode ultrasound technique to measure the displacement of the tricuspid ring which is only in the longitudinal direction. However, many conditions have been described where TAPSE do not reflect true RV function, which including patients after cardiac surgery, tetralogy of Fallot, and tricuspid regurgitation^21^. Furthermore, in patients with reduced ejection fraction heart failure may preserve TAPSE, hence, RV free-wall strain provides incremental prognostic information and improved risk stratification^22^. In these cases, new methods such as strain or strain rate of RV volumes deserve to get more attentions in future studies.

Interestingly, suppressed RV radial strain at the onset of hypoxic exercise and following HIIT was noticed. This is in contrast to the traditional viewpoint of enhancing longitudinal strain as the key contributor to overall RV contractility under RV overloading^6^. Regarding RV dilation, the reduced radial strain is considered to further increase wall tension. In this case, we believe that the significantly reduced hypoxic exercise-related PVR and dilated RV in HIIT are accommodations to overcome this situation. Briefly, these findings may represent a consequence of RV remodeling rather than dysfunction in healthy young men. In fact, our findings are partly similar to athletes’ heart characteristics, with a relative decrease in radial shortening with greater RV enlargement and better RVEF^23^.

The augmented RV radial strain rate in the HIIT group may indicate a greater RV contractile efficiency by homeometric autoregulation in response to the decreased radial strain. Strain rate is relatively more independent of heart rate, structure, and loading conditions than strain and diameter^24^. Hence, strain rate might better reflect the training responses and appears to be the more accurate parameter in myocardial contractility, especially during exercise^25^. In addition to the context of loading and structure, in some hypoxia-susceptible patients, RV dysfunction has been suggested to be caused by a direct negative inotropic effect of hypoxia on cardiac myocytes and decreased oxygenation^26^. Although the comparison of effects of normoxia and hypoxia on cardiac mechanics is not the main aim of this study, we focused on the fact that both RV diastolic and systolic functions were augmented when facing both exercise and loading stresses after 6 weeks of exercise training.

An elevated PVR is a well-known physiological response to hypoxia^27^. In this investigation, both HIIT and MICT reduced PVR and subsequently decreased RV afterload, thereby improving RVEF in resting conditions. The PVR and RVSP were further reduced during hypoxic exercise in HIIT, thus additionally lowering the afterload when the RV contracted. Previous studies demonstrated that exercise training upregulated endothelial eNOS expression in the pulmonary vasculum^28^. Hassel et al*.* further revealed that HIIT reduced the muscularization of pulmonary vessels and subsequently attenuated RV dysfunction in COPD mice^13^.

The fixed absolute exercise intensity used in hypoxic exercise is due to the concern about the influence of hypoxic exercise on an altered loading state by the different VO_2max_ after training. Therefore, our intention is to compare the relative change before and after the intervention rather than the absolute data. In addition, our previous study demonstrated that this protocol is feasible to clarify the LV mechanics during HIIT and MICT^29^.

The functions of the left ventricle (LV) and RV are intimately linked. An acute increase in PVR led to a change in LV diastolic function but without intrinsic alteration in contractility; nevertheless, when LV end diastolic pressure is increased due to LV systolic dysfunction, PVR becomes raised^30^. In contrast to patients with cardiopulmonary disease or pulmonary hypertension, in healthy subjects, overt LV diastolic dysfunction was not observed during acute hypoxia exposure^31^. A previously study indicated that hypoxia in normal subjects is associated with altered diastolic function of both ventricles, improved LV systolic function, and preserved RV systolic function^32^. However, in most cases, the elevated PVR was due to functional or structural abnormalities of the pulmonary vascular bed. Endurance training is well demonstrated that may improve the response to acute hypoxia by increasing efficacy of gas exchange and accompanied by smaller increases in PVR and PVR/CO on acute exposure to hypoxia, indicating that exercise training attenuates the vasocontraction, hence lowering RV work^33^. Although the superior effects of HIIT on contractile efficiency of the right heart may be due to more effectively improved LV performance in sedentary males as our previously work represented^29^. The greater venous return after improving LV performance may be a consequence of lower pulmonary resistance. The present study, however, is unable to provide further insight into these other possible mechanisms. The clinical relevance of these observations needs to be further investigated.

Although the average CO were similar during HIIT and MICT, the high intensity periods in HIIT provides more stimulations and allows for greater physiological stimulus and adaptation than MICT for cardiorespiratory fitness or cardiometabolic processes^34^. We have indicated that instead of MICT, HIIT can effectively improve the LV mechanics during exercise by increasing both contractile and diastolic functions^29^. HIIT also increases LV preload by enlarged end-diastolic internal dimension with increasing E/A ratio, thereby raising stroke volume (SV) during exercise^29^. These data have displayed that HIIT might be more efficient than MICT in physiological cardiac remodeling, and the changed intraventricular dimension were associated with cardiac mechanical functions. In present study, we further indicated more dominant RV structural alteration in the HIIT. We speculate the higher CO demand in HIIT may elicit a greater relative increase in pulmonic systolic pressure and wall stress, furtherly contribute to facilitate cellular edema and cardiomyocyte stimulation in the cardiac wall^35^. In previous study, only 2 weeks of HIIT induce changes in RV glucose metabolism, volumes, and ejection fraction, which precede exercise-induced hypertrophy of RV^36^. In addition, a significant enhanced eNOS expression were found following a HIIT in a rat model as well^28^. This presumption can be strengthened with findings by Park and Omi^37^, who stated an increase in pulmonary eNOS levels depending on the type of exercise performed^38^. However, even though HIIT is associated with multiple benefits, yet large-scale high-quality studies are still required to reaffirm and expand these findings.

### Clinical prospection

As increasing amount of people travel to high-altitude environments per year for work or recreation, reductions in the partial pressure of ambient oxygen initiate a cascade of physiologic responses which place unique stressors on the cardiovascular (mainly on the RV) and pulmonary systems. Our study demonstrated that exercise training may further diminished hypoxia -related pulmonary vascular reserve impairment, thus restoring exercise capacity in hypoxic, which was similar to the sildenafil-induced improvement in hypoxic exercise capacity^39^. Although our study does not validate the ranges but gives insight into relative changes after hypoxia exposure. The comprehensive functional cardiac testing including exercise stress testing in normoxia as well as hypoxia prior to altitude exposure has the potential to uncover impaired functional reserve of the cardiovascular system and to identify silent pathology. Consequently, it might help to reduce high-altitude associated complications like acute RV failure. In addition, the pulmonary pressure-reducing and RV-preserving effect observed only with HIIT encourages further investigation of this alternative training approach in other models and in patients as a potentially more optimal exercise regimen for PAH or COPD. If available, such exercise strategy could have tremendous clinical implications because RV function is a major independent prognostic determinant of those patients. Therefore, our finding still needs to be further investigated in different stage among different diseases.

### Limitations of the study

As observed in other investigations, the number of men who are young, healthy, and sedentary is limited. Thus, additional clinical evidence is required to extrapolate the present results to patients with abnormal cardiovascular systems, such as those with pulmonary hypertension or right heart failure, and to analyze potential sex differences^40^.

Because of the thin walls of the RV, the image quality might have highly influenced the accuracy of our detection. Although our test–retest reliability indicates good imaging quality, it is still important to note the limitations of 2D-STE^41^.

The noninvasive estimation of PVR might not have obtained true absolute data. However, it has been reported that the estimate has an error margin of < 10% relative to the real pressure^42^. Furthermore, using noninvasive echocardiography is more ethical than using invasive catheterization under dynamic conditions, with a much lower risk for the study participants^43^.

The MICT exercise volume is speculated to have been too low to exert a positive effect on cardiac hemodynamic adaptation. The plurality of the positive MICT results suggested that exercise training at least 5 days weekly up to six times daily for a period of at least 12 weeks is necessary^44^.

## Conclusion

Typically, right cardiac performance is assessed by echocardiography under normoxic environments, which displays only the chamber size and myocardial motion with a normal PVR. This study further contributes to a greater understanding of RV and RA mechanical responses to hypoxic stress following various exercise interventions by using 2D-STE under hypoxia exercise. The experimental results clearly demonstrate that HIIT with a dilated RV enhances RVEF by increasing the RA reservoir and conduit functions, enhancing RV radial strain rate and decreasing PVR. These findings provide novel insights into the superior effects of HIIT on contractile efficiency of the right heart during hypoxic exercise by simultaneously increasing preload and decreasing afterload, which might have important implications for exercise training in cardiopulmonary rehabilitation.

## Methods

### Study design

This is a single-blinded (outcomes assessor), parallel assignment, randomized control trial. After baseline testing, subjects were randomized in a 1:1:1 ratio to receive a 6-week exercise program consisting of either high intensity interval training (HIIT), moderate-intensity continuous training (MICT) or the control group (CTL) without engaging in any exercise. The investigation was performed in accordance with the principles of the Declaration of Helsinki and adheres to the Consolidated Standards of Reporting Trials (CONSORT) guidelines. The protocol was approved by the Institutional Review Board (IRB) of the Chang Gung Memorial Hospital in Taiwan (104-9615A3) and the trial was registered at the ClinicalTrials.gov (NCT04815460; registered by 25/03/2021). All participants provided fully-informed, written consent and the trial is now closed.

Participants were allocated a unique trial identifier number based upon sequence of recruitment. Due to the small sample size in each arm, permuted-block randomization was used via computer-generated random numbers to ensure equal sample size^45^. The study subjects were enrolled and assigned to their respective interventions by an unblinded project manager. Due to the nature of the intervention neither subjects nor staff could be blinded to allocation. The outcomes assessor and data analyst were blinded after study completion by having the subjects' intervention group information coded. Once the trial was initiated, no changes were made to the study design or outcome measures.

The primary outcome was the change of HIIT versus MICT on right cardiac mechanics during semiupright bicycle exercise under hypoxic conditions (i.e., 50 watts under 12% FiO2 for 3 min) as measured by two-dimensional speckle tracking echocardiography (2D-STE) at baseline and following the 6-week study period. The secondary outcomes were the conventional echocardiographic parameters, structure of RV and cardiopulmonary fitness at baseline and after interventions.

The sample size was estimated based on our previous literature and a pilot study^29^, which examined the effects of exercise training on similar outcome measures. The power analysis (G*Power: version 3.1., University of Dusseldorf, Germany) determined that the number of participants to detect significant changes was 18 per group, based on 1-β = 0.89, two-sided α = 0.05, and the effect size from a preliminary study = 0.7. The p-values were interpreted with care, as descriptive weights of evidence rather than as confirmatory claims. Furthermore, the cardiac mechanic data obtained from this investigation exhibited high values of a post hoc statistical power, from 0.853 to 1.000.

### Participants

A total of 56 sedentary males were assessed for eligibility from Chung Gang University (Taiwan). Of those, 2 were excluded as they declined to participate. Remaining participants (n = 54) were randomly allocated to each group (Supplementary Fig. S2). We recruited males who were nonsmokers; did not take medications or vitamins; did not have any cardiopulmonary/hematological risks; and, most importantly, had a sedentary lifestyle (without regular exercise; exercise frequency ≤ once weekly, duration < 20 min). Informed consent was obtained from all subjects after the experimental procedures were explained. Once all baseline data were collected, participants were randomized into one of the three groups: the HIIT (n = 18), the MICT (n = 18) or the CTL (n = 18). All subjects arrived at the testing center at 9:00 AM to eliminate any possible circadian effect.

### Training protocols

As our previously study, both the HIIT and MICT groups executed exercise training on a stationary bicycle ergometer (Corival 400, Lode B.V., Netherlands) 5 times a week for 6 weeks^29^. CTL participants kept their original diets and physical activity habits for 6 wk. The HIIT subjects warmed up for 3 min at 30% of oxygen uptake reserve (i.e., 30% × (peak VO_2_ − resting VO_2_) + resting VO_2_) before performing five exercise cycles, each cycle included 3 min at 80% oxygen uptake reserve with a 3-min active recovery period at 40% oxygen uptake reserve. The MICT group had the similar warm-up and cooldown protocols as the HIIT group except that the training period was 30 min at 60% of oxygen uptake reserve. Both training protocols were isovolumetric with the same duration (i.e., HIIT exercise volume: (3 min × 40% of oxygen uptake reserve + 3 min × 80% of oxygen uptake reserve) × 5 cycles = MICT exercise volume: (30 min × 60% of oxygen uptake reserve) × 1 cycle). Each subject used a heart rate (HR) monitor (Tango, SunTech Medical, UK) to obtain the assigned intensity of exercise. To reach the desired exercise intensity, the target HR was ensured by continuously adjusting the workload of the bicycle ergometer throughout the training period. The target HR was determined due to the heart rate reserve (%HRR), which is well considered to be equivalent to the % oxygen uptake reserve for exercise prescription purposes^46^. All subjects performed a cardiopulmonary exercise test (CPET) to exhaustion at the baseline, during which VO_2_ and HR were monitored to determine peak values. The peak HR data measured by the test are all reached or exceeded the age-based predicted maximal heart rate (HR_max_ = 220-age). Accordingly, the target HR of HIIT and MICT were calculated using the following equations:

1 $${\text{Peak HR}} = {\text{measured by cardiopulmonary exercise testing at the baseline}}$$

2 $$\% {\text{HRR}} = \% \left( {{\text{peak HR}} - {\text{resting HR}}} \right) + {\text{resting HR}}$$

3 $${\text{Target HR of HIIT}} = 3 - {\text{minute intervals at }}40\% {\text{ HHR and }}80\% {\text{ HRR}}$$

4 $${\text{Target HR of MICT}} = {\text{sustained }}60\% {\text{ HRR}}$$

The groups were asked to record their daily activities and nutritional intake using the short form of the International Physical Activity Questionnaire^47^ and a Written Diet Record^48^, respectively. Subjects were asked to refrain from regular extra exercise until the end of the study. Moreover, all subjects completed the experiments with a participant compliance rate of 100% and without any exercise-induced adverse effect. Additionally, every trainer are licensed physical therapists although subjects were trained by different trainers. In addition, the target HR is an objective parameter, which does not be influenced by the subjective perception from the trainer.

### Cardiopulmonary exercise test (CPET)

To assess aerobic capacity, a CPET on a cycle ergometer (Corival 400, Lode B.V., Netherlands) was performed 2 days before and after the intervention, which is sufficient to recover cardiac output following hypoxic exercise^49^. The data was collected by two experienced physicians. One physician was responsible for the pre-intervention tests and the other was responsible for the post-intervention data. Neither of them was blinded to know the group assignment or experimental timepoint. The maximal endpoint and the ventilatory threshold were decided by other two experienced physicians. If these two physicians had discrepantly consensus, a third physician's opinion was added. All subjects underwent exercise using a face mask to measure respiratory minute volume (V_E_), oxygen consumption (VO_2_), and carbon dioxide production (VCO_2_) breath by breath using a computer-based system (MasterScreen CPX, CareFusion, USA). After a 5-min baseline resting period, a 2-min warm-up period (60 rpm, unloaded pedaling) was initiated, followed by incremental work (30 W elevation for each 3 min) until exhaustion (i.e., progressive exercise to maximal oxygen consumption (VO_2max_)). The criteria used to define VO_2max_ were as follows: (i) the level of VO_2_ increased by < 2 mL/kg/min over at least 2 min; (ii) HR exceeded its predicted maximum; (iii) the respiratory exchange ratio exceeded 1.2; and (iv) the venous lactate concentration was > 8 mM. These criteria were consistent with the American College of Sports Medicine guidelines for exercise testing^50^. During CPET, continuous monitoring of 12-lead electrocardiography, blood pressure, and pulse oxygen saturation was performed. The SV, CO and TPR data was measured by the non-invasive cardiac output monitoring (NICOM, Cheetah Medical Inc., Portland, OR, USA). Some references have enough correlation between NICOM versus that measured from the Swan-Ganz catheter by thermodilution method^51^. In addition, the ventilation threshold was determined when V_E_/VO_2_ increased without a corresponding increase in the V_E_-to-VCO_2_ ratio, end-tidal PO_2_ increased without a decrease in end-tidal PCO_2_, or a deviation from linearity for V_E_.

### Conventional echocardiography

A standard echocardiographic examination according to the American Society of Echocardiography guidelines was performed at each stage^52^. Each subject underwent echocardiography 4 days before and after the intervention in an air-conditioned normobaric hypoxia chamber (Colorado Mountain Room, USA). The hypoxia chamber was maintained at a temperature of 22 °C ± 0.5 °C with a relative humidity of 60% ± 5%; a CO_2_ scrubber eliminated CO_2_ in the air (< 3500 ppm), and the O_2_ concentration was set at 12%, which corresponded to an altitude of 4460 m. All subjects were positioned at a 30° semiupright position oriented in a left lateral 60° semisupine position and secured to the echocardiography table (Angio with Echo Cardiac Stress Table, Lode B.V., Netherlands). The parameters were measured using the Siemens ACUSON SC2000 ultrasound system (Siemens Healthineers, Germany) with the 4V1C probe (4.5 MHz). Images of subjects with regular breathing patterns and no breath holding were captured at end expiration. The RV outflow tract (RVOT) was obtained from a parasternal short-axis base view, and the flow immediately proximal to the pulmonary artery valve during systole was detected to calculate both maximal velocity and pulsed-wave blood velocity time integral (VTI) (Supplementary Fig. S3). Doppler imaging was used to measure peak tricuspid annular velocities through the cardiac cycle in early diastole (E’) and diastolic transmitral blood flow velocities for peak early (E) fillings. Tricuspid annular plane systolic excursion (TAPSE) was measured by placing an M-mode cursor through the tricuspid annulus and measuring peak systolic motion. The RA pressure was estimated from the inferior vena cava size during inspiration and during forced inhalation at rest. The inferior vena cava diameter was measured just proximal to the entrance of the hepatic veins. Pulmonary vascular resistance (PVR) was calculated using the formula PVR = ([tricuspid regurgitation velocity/RVOT VTI] × 10 + 0.16), which has shown a good correlation with invasively derived PVR^53^. All data were recorded over three cycles, and the averages were calculated. RV basal cavity diameter (RVD1), mid-cavity diameter (RVD2), RV longitudinal diameter (RVD3), and RV area at end-diastole and end-systole were evaluated in the modified apical four-chamber view, as shown in Supplementary Fig. S3. All measurements were independently recorded from three independent image frames, enabling reliable quantification.

### Two-dimensional Speckle-tracking echocardiography (2D-STE) of hypoxic exercise

2D-STE was immediately performed after the conventional data were collected completely under hypoxic conditions (12% FiO_2_) as previously described^29^. Hypoxic resting images were acquired after the subject was placed in the aforementioned position for 10 min. The hypoxic exercise images were conducted using semirecumbent cycling with a 50-Watt resistance for 3 min and acquired at the third minute of cycling to ensure that subjects had reached a steady-state HR (i.e., HR changes < 10 bpm within 10 s and < 110–120 bpm)^54^. Three consecutive cardiac cycles were evaluated for each acquisition.

A modified apical four-chamber view was used to assess 2D-STE longitudinal and radial parameters of the RV and RA. Briefly, after manual tracing, the end-systolic RV endocardial border, a region of interest, was automatically generated; its width and position were manually readjusted to include the entire myocardial wall when it showed poor-quality tracking by visual assessment. The software automatically divides the RV into a 6-segment model as a more robust analysis recommend by Muraru et al*.*^40^, whereas the RA was automatically divided into a 3-segment model. The RV strain and strain rate were calculated using the average peak segmental values displayed by the software using a 6-segment model. The compliance rate of this study was 100%, and no subject was excluded due to inadequate images.

### Volumetric analysis in RA function

**Figure 4:**
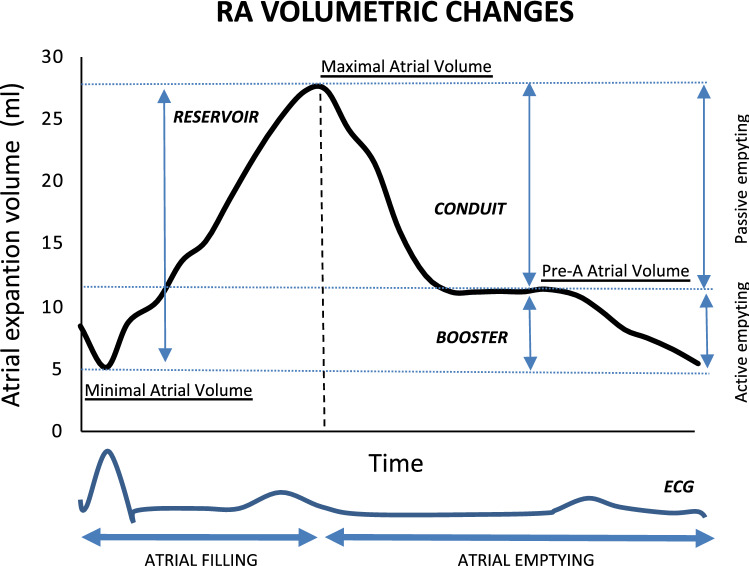
Schematic RA time-volume curve. RA time-volume curve with the assessment of parameters of atrial function. Reservoir, conduit, and booster volumes are calculated from atrial volumes at special time points. Reservoir function = Vmax-Vmin; Conduit function = Vmax-Vpre-A; Booster function = Vpre-A-Vmin; RV Vmax, maximum RV volume; RV Vmin, minimum RV volume; Pre-A: preatrial contraction.

RA volumes were assessed offline using semiautomatic strain software (Siemens ACUSON SC2000 ultrasound system, Siemens Medical Solutions USA Inc., Mountain View, CA) on dedicated 2D-STE sets in the apical four-chamber view. The border-tracing process was similar to the abovementioned 2D-STE protocol. RA maximum volume (RA_max_) was detected at the end of LV systole just before mitral valve opening, and RA minimum volume (RA_min_) was acquired at the end of LV diastole just after mitral valve closure. Atrial function is most often assessed using 2D volumetric analysis, such as reservoir, conduit, and booster pump functions. The volume immediately before atrial contraction (onset of P wave) is denoted as RA_pre-a_, which represents the preload before atrial contraction. Figure 4 shows the schematic RA time-volume curve.

(1) **Reservoir volume**: the filling or expansion volume, calculated as RA_max_ – RA_min._

(2) **Conduit volume**: the passive emptying volume from venous return during early ventricular diastole, calculated as RA_max_ – RA_pre-a_.

(3) **Booster volume**: the RA stroke volume (SV), calculated as RA_pre-a_ –RA_min._

This study used one licensed echocardiogram technician to perform the collection among conventional echocardiography, 2D-STE and RA volumetric analysis data. And the data collector was isolated from the data analytic specialist. Analysis was performed offline by one echocardiographer, who was blinded to the group allocation and image sequence, using semiautomatic strain software (ACUSON SC2000 system, Siemens Healthineers, Germany).

### Test–retest reliability

A subgroup (n = 20) was assessed for test–retest variability in RV radial and longitudinal strains. Each participant had two separate echocardiograms using the same set of 2D-STE images under normoxic conditions that were approximately 24 h apart to reduce the impact of physiological variation^55^. The echocardiographer was blinded to the original images and used a standard echocardiographic protocol for each acquisition. Offline analyses were randomized by the same echocardiographer and performed using available software (Siemens ACUSON SC2000 ultrasound system, Siemens Medical Solutions USA Inc., Mountain View, CA).

### Statistical analysis

Quantitative data were expressed as the mean ± SEM. Data analysis was performed using IBM SPSS Statistics V22.0. Experimental results were analyzed by repeated-measure ANOVA and Bonferroni post hoc tests to compare aerobic capacity and cardiac mechanics at the beginning of the study and after 6 weeks of intervention. Linear regression analyses were performed using Pearson’s method to assess univariate associations between echocardiographic data. Intra-reproducibility was assessed using the intraclass correlation coefficient (ICC), coefficient of variance (CV), and Cronbach alpha value^56^. The threshold for statistical significance was set at *P* < 0.05. The datasets generated during and/or analyzed during the current study are available from the corresponding author on reasonable request.

## Supplementary Information

Supplementary Information.

## Abbreviations

%HRR: Heart rate reserve
2D-STE: Two-dimensional speckle-tracking echocardiography
CO: Cardiac output
CPET: Cardiopulmonary exercise test
CTL: Control group
FAC%: Fractional area change
FiO_2_: Fraction of inspiration oxygen
HIIT: High-intensity interval training
LV: Left ventricle
MICT: Moderate-intensity continuous training
post-: After the intervention
pre-: Before the intervention
PVR: Pulmonary vascular resistance
RA: Right atrial
RV: Right ventricular
RVD1: RV basal cavity diameter
RVD2: RV middle cavity diameter
RVD3: RV longitudinal diameter
RVEF: Right ventricular ejection fraction
RVOT: Right ventricular outflow tract
RVSP: Right ventricular systolic pressure
SR: Strain rate
SV: Stroke volume
TAPSE: Tricuspid annular plane systolic excursion
TPR: Total peripheral resistance
VCO_2_: Carbon dioxide production
V_E_: Respiratory minute volume
VO_2_: Oxygen consumption
VO_2__max_: Maximal oxygen consumption
VTI: Velocity time integral
