# Supplementary material for: A randomized controlled trial of enhancing hypoxia-mediated right cardiac mechanics and reducing afterload after high intensity interval training in sedentary men
Source: Sci Rep. 2021 Jun 15;11:12564. doi: 10.1038/s41598-021-91618-0 (PMC8206117; doi:10.1038/s41598-021-91618-0)
Supplement: Supplementary file 1 — Supplementary Information. [file 41598_2021_91618_MOESM1_ESM.docx]

**Supplementary Data**

**A Randomized Controlled Trial of Enhancing Hypoxia-Mediated Right Cardiac Mechanics and Reducing Afterload After High Intensity Interval Training in Sedentary Men**

Yu-Chieh Huang, PhD^1^; Chih-Chin Hsu, MD, PhD^2^; Tieh-Cheng Fu, MD, PhD^2^; Jong-Shyan Wang, PhD^2,3,4*^

^1^ Department of Physical Therapy, College of Medical and Health Science, Asia University, Taichung, Taiwan

^2^ Heart Failure Center, Department of Physical Medicine and Rehabilitation, Keelung Chang Gung Memorial Hospital, Keelung, Taiwan

^3^ Healthy Aging Research Center, Graduate Institute of Rehabilitation Science, Medical Collage, Chang Gung University, Taoyuan, Taiwan

^4^ Research Center for Chinese Herbal Medicine, College of Human Ecology, Chang Gung University of Science and Technology, Taoyuan, Taiwan

**Supplementary Table S1**

**Supplementary Figure S1**

**Supplementary Figure S2**

**Supplementary Figure S3**

| **Supplementary Table S1: Reliability data of RV strain variables** | | | | | |
| --- | --- | --- | --- | --- | --- |
|  | Intra-observer | | | | |
|  | ICC | 95% CI | CV% | Cronbach Alpha | r |
| RV radial strain, % | 0.89 | 0.74-0.95 | 7.9 (7.6-8.2) | 0.94 | 0.9 |
| RV longitudinal strain, % | 0.93 | 0.83-0.97 | 5.7 (5.2-6.2) | 0.96 | 0.95 |

ICC: intraclass correlation coefficient

CI: Confidence interval

CV%: Coefficient of variation

**Supplementary Figure S1**

**Fig. S1: Test-retest comparisons of** (A) RV radial strain correlation dot plot, (B) RV longitudinal strain correlation dot plot, (C) RV radial strain Bland–Altman plots and (D) RV longitudinal strain Bland–Altman plots for 2D-STE in intra-observer reproducibility. (n=20)


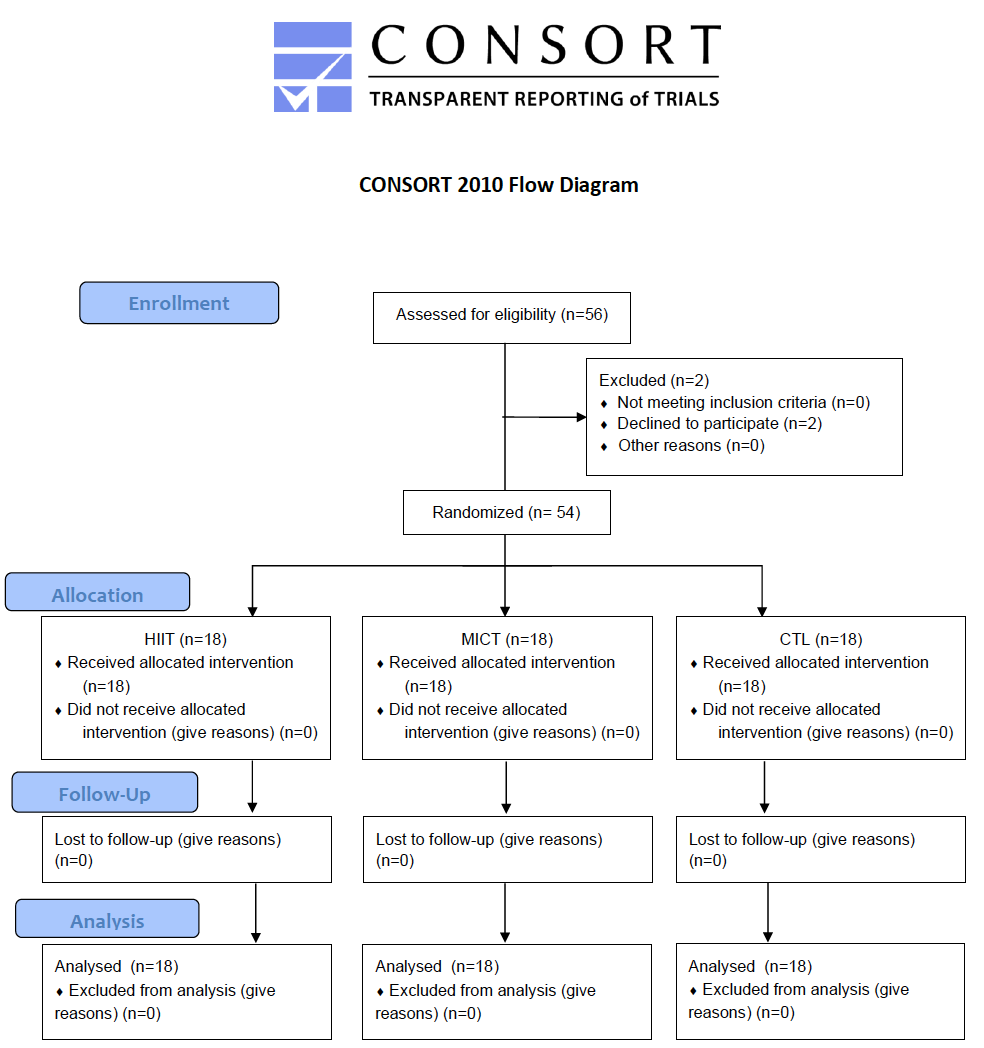
**Supplementary Figure S2**

**Fig. S2: A flow chart for the data collection for the intervention and control group.** This chart is reported according to Consolidated Standards of Reporting Trials (CONSORT) guidelines.

**Supplementary Figure S3**

**Fig. S3: An example of a single screenshot of the echocardiogram in a subject showing landmarks. Quantification of** (A) RV area in end-diastole, (B) RV area in end-systole, (C) RV cavity diameters in end-diastole, (D) RV cavity diameters in end-systole and (E) RVOT was obtained from a parasternal short-axis base view to detect the flow immediately proximal to the pulmonary artery valve and to calculate the pulsed-wave blood VTI. **RVD1:** basal cavity diameter, **RVD2:** mid cavity diameter, **RVD3:** longitudinal cavity diameter, **RVOT:** right ventricular outflow tract, **RA:** right atrium, **LA:** left atrium, **AoV:** aortic valve, **PV:** pulmonary valve, **PA:** pulmonary artery, **VTI:** velocity time integral.
